# The association between family and community social capital and health risk behaviours in young people: an integrative review

**DOI:** 10.1186/1471-2458-13-971

**Published:** 2013-10-19

**Authors:** Kerri E McPherson, Susan Kerr, Antony Morgan, Elizabeth McGee, Francine M Cheater, Jennifer McLean, James Egan

**Affiliations:** 1 Institute for Applied Health Research, School of Health & Life Sciences, Glasgow Caledonian University, Cowcaddens Road, Glasgow G4 0BA, UK; 2 GCU London, 40 Fashion Street, Spitalfields, London E1 6PX, UK; 3 School of Nursing Sciences, Faculty of Medicine and Health, University of East Anglia, Norwich Research Park, Norwich NR4 7TJ, UK; 4 Glasgow Centre for Population Health, 1st Floor, House 6, 94 Elmbank Street, Glasgow G2 4DL, UK

**Keywords:** Family social capital, Community social capital, Children, Adolescents, Health risk behaviours, Wellbeing, Health

## Abstract

**Background:**

Health risk behaviours known to result in poorer outcomes in adulthood are generally established in late childhood and adolescence. These ‘risky’ behaviours include smoking, alcohol and illicit drug use and sexual risk taking. While the role of social capital in the establishment of health risk behaviours in young people has been explored, to date, no attempt has been made to consolidate the evidence in the form of a review. Thus, this integrative review was undertaken to identify and synthesise research findings on the role and impact of family and community social capital on health risk behaviours in young people and provide a consolidated evidence base to inform multi-sectorial policy and practice.

**Methods:**

Key electronic databases were searched (i.e. ASSIA, CINAHL, Cochrane Database of Systematic Reviews, Cochrane Central Register of Controlled Trials, Database of Abstracts of Reviews of Effects, Embase, Medline, PsycINFO, Sociological Abstracts) for relevant studies and this was complemented by hand searching. Inclusion/exclusion criteria were applied and data was extracted from the included studies. Heterogeneity in study design and the outcomes assessed precluded meta-analysis/meta-synthesis; the results are therefore presented in narrative form.

**Results:**

Thirty-four papers satisfied the review inclusion criteria; most were cross-sectional surveys. The majority of the studies were conducted in North America (n=25), with three being conducted in the UK. Sample sizes ranged from 61 to 98,340. The synthesised evidence demonstrates that social capital is an important construct for understanding the establishment of health risk behaviours in young people. The different elements of family and community social capital varied in terms of their saliency within each behavioural domain, with positive parent–child relations, parental monitoring, religiosity and school quality being particularly important in reducing risk.

**Conclusions:**

This review is the first to systematically synthesise research findings about the association between social capital and health risk behaviours in young people. While providing evidence that may inform the development of interventions framed around social capital, the review also highlights key areas where further research is required to provide a fuller account of the nature and role of social capital in influencing the uptake of health risk behaviours.

## Background

A significant proportion of premature adult deaths are considered to have their antecedents in late childhood and adolescence [1]. It is during this time that young people generally experiment with and establish health behaviours known to result in poorer health outcomes in adulthood, including smoking, alcohol and illicit drug use and sexual risk taking [2]. As might be expected, exposure to health risk behaviours increases as the adolescent years advance, with the period between 11 and 15 years being of particular significance [3-5].

Understanding the trajectory and the underlying determinants of health risk behaviours in young people is essential if interventions are to be developed to reduce uptake. The family provides the first context for infants and very young children and families provide a vehicle through which children become part of their local communities; however, it is generally accepted that, as children mature, the wider environment and social interaction take on a larger role [6-8]. This wider social context, in addition to the influence of family, is therefore crucial to our understanding of the ways in which adolescents experience and manage their own health and wellbeing, including how they access, generate and mobilise ‘social capital’ [9,10].

Social capital has been described as a resource for societies [11-13]. While it has its roots in the work of sociologists such as Durkheim [14], its acceptance as a concept which has the potential to further articulate the relationship between health and its broader determinants, stems from the work of Pierre Bourdieu [15], James Coleman [16] and Robert Putnam [17]. Each of these theorists describes social capital through a different disciplinary lens and this has generated considerable debate within the literature about how social capital should be conceptualised and measured [18-20]. However, despite its multi-dimensionality, a common thread running through the various definitions of social capital relates to the importance of positive social networks and relationships in bringing about social, economic and health development among different groups, hierarchies and societies [21,22].

Theoretical consideration of social capital as a resource for the health and wellbeing of young people first appeared in the literature in the 1990s [9,17,18,20,21], with the empirical evidence base accumulating over the past 10 years. Yet, to date, there have been few attempts to consolidate the evidence in such a way as to provide a meaningful framework upon which social capital interventions can be built to promote protective environments for young people, particularly in the context of their health and wellbeing [23,24]. Moreover, it has been argued that, as a construct developed within an adult framework, traditional definitions of social capital may be inadequate in the context of young people. Young people’s lives may differ from adults’ in terms of social spaces and social connections [21,25] and developments in the sociology of childhood highlight the importance of children’s agency, autonomy and involvement in the health process; it is therefore important to acknowledge that they are capable of generating and using social capital in their own right [26]. For example, schools are often not included in definitions of social capital but they are an important aspect of community for young people, representing places where social networks are formulated and exploited for support [27].

In one of the few attempts to synthesise the literature, Ferguson “explored how social capital had been conceptually and operationally defined as a predictor variable in empirical research on individual and collective wellbeing, especially in relation to children and youth” [24]. Acknowledging the importance of bonding relationships that exist within families, and relationships that bond and bridge families to local communities, Ferguson sought to identify key indicators of both family and community social capital (e.g. the quality of parent–child relations, extended family support, social support networks and the quality of school) and considered their influence on wellbeing. However, while this review highlights the importance of both family and community social capital in the context of young people, the construct of wellbeing was ill-defined and the outcomes indicators broad (e.g. physical health, educational attainment). Importantly, for our purpose, Ferguson’s review did not focus on the association between social capital and health risk behaviours.

More recently, a review undertaken by Vyncke and colleagues [20] explored the role that neighbourhood social capital may play in levelling the social gradient in the health and wellbeing of children and adolescents. While this paper makes an important contribution in the field, the outcomes of interest were behavioural problems, self-esteem and cognitive abilities, rather than health risk behaviours, and the synthesised evidence was limited to neighbourhood social capital (but excluding schools). This focus on neighbourhood means that the influence of family social capital was not explored.

This integrative review is, therefore, the first attempt to systematically identify and synthesise published empirical literature exploring the role and impact of both family and community social capital on health risk behaviours in young people. Cognisant of the various theoretical traditions and definitions within the social capital literature we adopted an inclusive and pluralistic approach drawing on the work of a broad range of theorists; including, but not limited to, Bourdieu, Coleman and Putnam. Given the focus on young people, however, the concepts of family social capital (FSC) and community social capital (CSC) have been used to frame the presentation of the results.

## Methods

This paper forms part of a larger review, which explored the association between social capital and a range of psychosocial indicators of health and wellbeing (e.g. mental health and problem behaviours and health promoting behaviours). The method for the larger review is published in full in the final report [23] and this includes the single search strategy developed to capture literature from across the range of psychosocial health and wellbeing outcomes. We present here the elements of the method directly relevant to collection and analysis of the health risk behaviour data.

### Criteria for considering studies for inclusion

#### Types of studies

Studies were included if they were published, peer-reviewed and described primary empirical research that was quantitative, qualitative or had employed mixed methods.

#### Types of participants

Studies were required to have focused on children and/or adolescents. Scoping of the literature revealed inconsistencies in the ways that authors defined children and adolescents so we adopted a pragmatic approach, guided by the WHO’s definition of adolescence [28]. Samples where the majority were 10–19 years old were described as ‘adolescents’ and samples where the majority were 5–10 years old were described as ‘children’. We also included ‘mixed age group’ samples.

We included studies where the data had been collected directly from the young person and where the data about the young person had been reported by a relevant other (e.g. parent, teacher or other professional).

#### Types of social capital

As noted above, we adopted a pluralistic approach to the conceptualisation of social capital but we were guided by Ferguson’s findings as a framework for categorising indicators of family and community social capital. Thus, studies were included if they explored the role and impact of social capital at the family and community level. The elements of FSC included: family structure (e.g. number of parents present in the household), the quality of parent–child relations (e.g. parent–child communication), adult interest in the child (e.g. parental involvement with school), parent’s monitoring of the child (e.g. perceptions of parental monitoring/control) and extended family support and exchange (e.g. perceptions of extended family support). The elements of CSC included: social support networks (e.g. peer support), civic engagement in local institutions (e.g. volunteering), trust and safety (e.g. trust in others), religiosity (e.g. attendance at religious services), the quality of the school (e.g. school cohesion and relationship between teachers and pupils) and the quality of neighbourhood (e.g. neighbourhood cohesion and social control). In addition, we included studies that employed a composite measure of FSC and/or CSC and studies where, although the indicator did not fit within the definition above, the author(s) explicitly described their work as FSC (e.g. family cohesion) and/or CSC (e.g. peer role models) and we refer to this as ‘other measure’.

#### Types of outcomes

Studies were included if they assessed individual-level health risk behaviours, including: alcohol use/misuse; smoking/tobacco use; illicit drug use; sexual health behaviours; and general health risk taking, where a composite health risk behaviour score was calculated to assess risk across a number of different domains (e.g. smoking, alcohol and drug use). Studies were only included where health risk behaviours were conceptualised and/or measured as outcome variables.

### Search strategy

#### Data sources

In April 2012, nine electronic bibliographic databases were searched for relevant published empirical literature: ASSIA, CINAHL, Cochrane Database of Systematic Reviews, Cochrane Central Register of Controlled Trials, Database of Abstracts of Reviews Effects, Embase, Medline, PsycINFO, and Sociological Abstracts.

Following the electronic database searches, the reference lists of retrieved articles were examined for further studies. The web-sites of organisations and groups conducting research on the health and wellbeing of children and adolescents, and/or research in the field of social capital, were also searched for published empirical literature (e.g. the Centre for Research on Families and Relationships and the WHO).

#### Search terms and delimiters

Scoping of relevant electronic databases helped identify the most appropriate search terms and a single search strategy was developed to capture literature from across the range of psychosocial health and wellbeing outcomes, including health risk behaviour terms. The search strategy included both index terms (i.e. thesaurus and subject headings) and free text keywords. Social capital-relevant search terms were combined with health and wellbeing outcome-relevant search terms. The search strategy was tailored to optimise its sensitivity within each database [29] and the PsycINFO search strategy is presented in Additional file 1.

Our searches were limited to literature published between January 1990 and April 2012 and to English language-only. *RefWorks* was used to store the results of each of the searches and to identify duplicates.

### Data collection and analysis

#### Selection of studies

After duplicates were removed, the title and abstract of each retrieved study were screened independently by two members of the research team, rejecting any that did not fit the inclusion criteria described previously. If no abstract was available, or the abstract did not contain sufficient detail, the study was retained for full text review. Again, the full text was reviewed against the inclusion/exclusion criteria by two independent reviewers. At both stages, few discrepancies were identified between the reviewers and any that did arise were resolved through discussion.

#### Data extraction

The full text of articles assessed as meeting the inclusion criteria was sourced for data extraction. A review-specific data extraction tool was developed to enable the extraction of data generated using a range of different research designs [23]. The data extracted included: the context of the study, such as geographical location and year(s) of data collection; the aims and purpose of the study; methodological considerations, such as design, participants and data collection methods; the main findings; and, the strengths and limitations of the study. Data were extracted from each of the studies by two reviewers who worked independently. At all stages, disagreements were resolved through discussion, involving a third reviewer if necessary.

#### Quality appraisal

Concurrently with data extraction, each reviewer assessed the methodological quality of the studies using a study-specific quality appraisal tool (QAT). The QAT was developed drawing on published guidance [30] and unpublished quality appraisal tools used previously by the team [23]. Made up of 11 criteria, each item was scored on a three-point scale (0=weak; 1=moderate; 2=strong), giving a possible range of scores from 0 to 22 for each paper. Disagreements were resolved through discussion, involving a third reviewer if necessary.

Upon consensus, each study was awarded one of three quality ratings, studies scoring: 16–22 were awarded a ‘high quality’ rating; 8–15 were awarded a ‘moderate quality’ rating; and, 0–7 were awarded a ‘low quality’ rating. The ratings for each included study are presented in Additional file 2 (column 1). Studies were not excluded on the grounds of quality.

#### Data analysis and synthesis

The results are presented in narrative form. The majority of studies were surveys and the associated design issues (e.g. lack of control groups) and heterogeneity in the outcome measures precluded meta-analysis. Moreover, meta-synthesis was not possible because few qualitative studies met the inclusion criteria.

Instead, results are summarised and then synthesised using an approach similar to that originally described by Ramirez et al. [31]. That is, results are grouped into three categories: results that show a positive association between social capital and health risk behaviours (i.e. where social capital was associated with better outcomes and the results were statistically significant); results that show a negative association between social capital and health risk behaviours (i.e. where social capital was associated with poorer outcomes and the results were statistically significant); and, results where no association between social capital and health risk behaviours was identified (i.e. results were not statistically significant).

## Results

### Study selection

**Figure 1:**
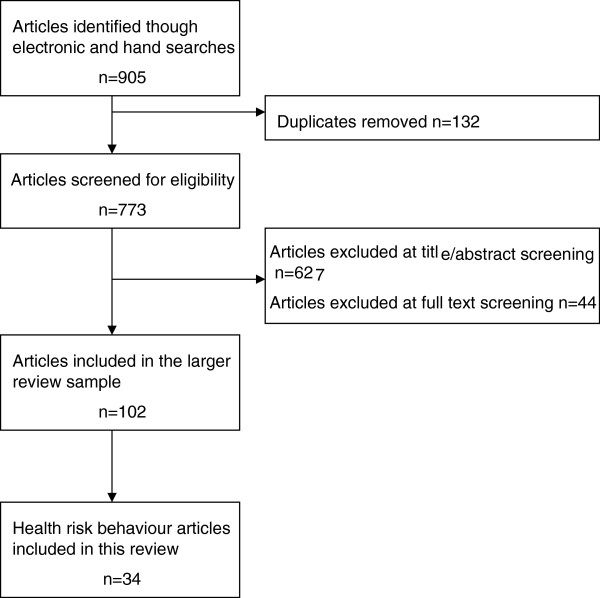
Flow diagram of search results.

In accordance with the PRISMA (Preferred Reporting Items for Systematic Reviews and Meta-analyses) guidelines [32] Figure 1 details the process from the initial search and screening through to final study inclusion. Following the searches and the removal of duplicates, 773 articles were screened against the inclusion/exclusion criteria. The majority (n=627) of the articles were excluded at the title and abstract stage and a further 44 were excluded at the full text stage. The primary reasons for exclusion were that the article did not fit: the definition of child/adolescent (n=389); the definition of health and wellbeing (n=115); the study design criteria (n=92); or the definition of FSC or CSC (n=73). A total of 102 articles were retained for inclusion across the health and wellbeing outcomes of the larger study and 34 of these included health risk behaviours and are the focus of this review. The outcomes of study selection processes are detailed in Figure 1.

### Description of studies

Descriptive information about each of the individual studies is presented in Additional file 2, this includes: the aims of the study, the geographical location and timing of the data collection, the study design, sample details, the health risk behaviour(s) assessed, the type of social capital assessed and the key results.

The health risk behaviours explored in the 34 studies were ordered into five outcome categories with 12 of the studies reporting on more than one of the outcomes. Eleven studies reported on tobacco use, 14 on alcohol use, nine on illicit drug use and 15 on sexual risk behaviours. Five of the studies reported on general health risk behaviours where the outcome was a composite score of risk across a number of behaviour domains. Six studies included at least one indicator of FSC and nine included at least one indicator of CSC. The remaining 19 studies included indicators of both FSC and CSC (see Additional file 2).

Thirty three of the studies were surveys, including 29 cross-sectional and four longitudinal surveys. The remaining study was a longitudinal cohort study. Over two thirds (n=22) were conducted in the United States, with further studies from Canada (n=3) and the UK (n=3). One was a multi-country study conducted in Europe and Canada and the remaining studies were conducted in China, El Salvador, Ethiopia, Japan and Switzerland. Eleven studies did not report when the data were collected, 14 studies began, and in some instances completed, data collection in the 1990s and nine began data collection after 2000 (see Additional file 2).

Across the 34 studies there was considerable variation in the reported sample sizes and some studies did not clearly articulate dropout rates. The number of participants also varied across the different analyses conducted in the studies and so we report on the maximum number of young people included in the analysis set; the largest being 98,340 and the smallest 61.

Thirty three studies reported on adolescent-only samples; two of these did not provide information on the ages of the adolescents but in the remaining 31 they ranged from 11 to 19 years. The final study reported on a mixed sample ranging from 8 to 18 years. Five of the studies did not report details of the sex of their sample. In the remaining 29, the percentage of female participants ranged from 44% through to 100%.

Twelve studies either did not report on the ethnicity, race or nationality of the sample or it was not possible to extract these data in a meaningful way. Fifteen studies described the majority as Caucasian, Non-Hispanic White, Non-Hispanic Caucasian or White and we grouped these under the single category ‘White’. Five studies described the majority as African American or Black and we grouped these under the single category ‘Black’ for reporting. In the two remaining studies the participants were described as ‘Swiss’.

The quality appraisal rating assigned to each of the studies is presented in Additional file 2 (column 1). The majority of the studies (n=22) were assessed as ‘high quality’, nine were assessed as ‘moderate quality’ and three as ‘low quality’.

### Tobacco use

Eleven studies (see Additional file 2) investigated the role and impact of FSC and/or CSC on tobacco use [33-43]. Seven studies explored FSC in the context of adolescent tobacco use and, in the majority, FSC was associated with better tobacco outcomes. Parent-adolescent relationships high in closeness, trust and nurturance were associated with less frequent, or non-use, of tobacco [34,41,42]. Two studies assessed the quality of the communication within parent-adolescent relationships; one [33] found positive communication between parent(s) and adolescent to be associated with lower levels of tobacco use but the other reported communication as being associated with increased use [41]. However, in the latter study parent-adolescent closeness diluted the risk associated with communication, such that relationships that were high in closeness and positive communication protected the adolescent against tobacco use.

Further supporting the protective role of positive intra-family relationships, two studies reported that adolescents from families that engaged in more joint family activities were less likely to use tobacco [35,37]. Parental monitoring had limited effect on tobacco use.

There was an inconsistent pattern of results across the six studies assessing social support networks but this may be accounted for, in part, by the type of assessment. Two studies assessed social support networks in terms of adolescents’ interactions with their friends/peers and both reported that increased connectedness and frequency of contact were associated with increased tobacco use [41,42]. Two further studies assessed participation in recreational clubs/groups, an indicator of wider social networks, and found that increased participation was associated with reduced tobacco use [33,43]. However, there was evidence to suggest that different types of groups/clubs may have differential impact; school-based groups had no association with tobacco use, youth clubs were associated with increased use and religious groups/clubs with decreased use [37,43].

Civic engagement, religious attendance and having peer/adult mentors also provide some insight into the reach of an adolescent’s networks. One study found all three were associated with reduced tobacco use [33] but this was not replicated across others. There was evidence that higher quality school [41], but not neighbourhood [39,40,42] environments were associated with lower levels of tobacco use.

In sum, social capital amassed through an adolescent’s interactions with other people is protective against tobacco use in some circumstances and creates a risk factor in others. In particular, positive relationships between the adolescent and other family members seem to be associated with reduced likelihood of the adolescent using tobacco. However, relationships that extend out of the family into the adolescent’s social sphere may in some instances create opportunities for risk behaviour. In contrast, the infrastructural support of the school environment appears to offer protection to adolescents in the context of tobacco use.

### Alcohol use

Fourteen studies (see Additional file 2) assessed the role and impact of FSC and/or CSC on alcohol use and all included mixed-sex samples of adolescents [34-39,42-49]. Eight studies explored the role and impact of FSC on alcohol use. Parent-adolescent relationships assessed as positive were associated with better outcomes [34,42,45,46,48]. Moreover, adolescents who reported that their families engaged in more joint activities (e.g. family mealtimes) reported fewer alcohol risk behaviours [35,36].

The pattern of association is less clear for parental monitoring. Two studies [38,42] reported that parental monitoring/control was unrelated to alcohol use while another found having a controlling father was associated with increased likelihood of using alcohol [37]. A further study found monitoring to be associated with reduced alcohol use for male adolescents only [34]. Having negotiated but unsupervised time with their peers was also associated with increased risk of males and females reporting alcohol use [34].

There was evidence that family relationships may have a differential impact across different groups of adolescents. There were conflicting results about the impact across the sexes with one study reporting that a positive parent-adolescent relationship was associated with reduced alcohol use in male adolescents only [34] and another reporting it was associated with reduced alcohol use in female adolescents only [48]. When broader family relationships were explored in the context of ethnicity a different pattern of association was evident across different ethnic groups; extended family support was beneficial for Mexican and Puerto Rican adolescents but a risk factor for Cuban adolescents [45] (see Additional file 2).

Half of the studies exploring social support networks and alcohol use failed to identify any association. Of the studies reporting an association, one reported that poorer quality peer relationships were associated with alcohol consumption cross-sectionally but not longitudinally [49], and another that peer connectedness was associated with increased use [42]. A third reported that, in general, club membership was protective but some club types were associated with increased odds of drunkenness (e.g. youth or sports clubs) and other types with decreased odds (e.g. cultural or religious clubs) [43]. Moreover, adolescents who reported they had peer, but not adult, role models were more likely to report abstinence [46]. There was some evidence that adolescents who engaged in active citizenship activities [37,44,46] and who had higher levels of trust in others [39,44] engaged in fewer alcohol risk behaviours. Three studies showed frequency of attendance at religious services to be associated with better outcomes but there was no consistent evidence showing a role for religious identity or personal importance of religion [44,46,47].

None of the studies explored the association between alcohol use and the quality of neighbourhood, but two found positive school attributes were associated with reduced alcohol use [37,42] and another that school cohesion was associated with better outcomes in female adolescents but poorer outcomes for males [48].

To summarise, in the context of alcohol use the protective effects of FSC and CSC are mixed. FSC offers the most consistent effects with positive relationships between young people and their parents being protective. On the other hand, parental monitoring/control appears to have little protective value and in some instances is associated with increased risk. The evidence for CSC is also inconsistent. That said, adolescents who participate in active citizenship and/or participate more frequently in religious services have better alcohol outcomes.

### Drug use

Nine studies (see Additional file 2) explored the role and impact of FSC and/or CSC on illicit drug use [34-36,38,42,44,46-48]. Six studies included indicators of FSC and a clear pattern was evident for family relationships. Adolescents who had a positive relationship with their parent(s) [34,42,46,48], and those from families that spent more time together [35], were less likely to report drug use. No role was identified for parental monitoring/control behaviours [34,38,42]. Linked to parental monitoring, adolescents who had negotiated unsupervised time with their peers reported more frequent marijuana use [34].

In the seven studies assessing CSC, there was limited and inconsistent evidence available about the role of social support networks. One study assessing participation in recreational clubs/groups, an indicator of wider social networks, reported a protective role [46], but another found peer connectedness was associated with poorer outcomes [42]. In contrast, adolescents who reported they had a peer, but not an adult, mentor had increased odds of reporting not using drugs [46].

Four studies found that adolescents with frequent attendance at religious services were less likely to report drug use [36,44,46,47]. There were, however, inconsistent findings in relation to the role of religious identity and the personal importance of religion [44,47]. No study explored the role of neighbourhood quality but there was some evidence that higher quality school environments offer some protection to students in relation to drug use [42,48], and one study reported that this was particularly important for females [48].

In sum, family relationships characterised by trust, support and nurturance appear to provide adolescents with assets that protect against drug use. In contrast, parental monitoring/control has little effect, although an indicator of a more *laissez faire* approach to monitoring, negotiated unsupervised time with peers, was associated with increased risk. In the context of CSC, adolescents accrue more protection from structural sources, religious attendance and school, than they do from their relationships with others, including their peers.

### Sexual health

Fifteen studies (see Additional file 2) explored the role and impact of FSC and/or CSC on sexual health outcomes [34,35,48,50-61]. In thirteen studies there was evidence that living with at least one biological parent was an important protective factor in the context of sexual health [50,56,57]. In addition, the absence of a father may be more important for some adolescents than others (e.g. younger adolescents) [50,56].

In terms of the parent-adolescent relationship, there was evidence that the *quality* of the relationship (e.g. trust and ease of communication) was associated with delay in first sexual experience [34,55] and the implementation of positive sexual behaviours, such as contraception use [34,57,59]. Conversely, in the only study to evaluate the *quantity* of parent-adolescent conversations about sex, adolescents reporting more frequent conversations were more likely to report having had sex [60]. The authors hypothesised that this increased frequency of parent-adolescent conversations may have been a consequence of the adolescent’s sexual behaviour rather than increased frequency of conversations increasing the likelihood of the adolescent having had sex.

Unlike the other risk behaviours, there was evidence that parental monitoring can have a positive impact on sexual health, being associated with increased likelihood of the adolescent reporting sexual abstinence [50,60,61] and, for sexually active adolescents, with positive sexual behaviour such as condom use [34,50]. Negotiated unsupervised time with peers, on the other hand, was associated with increased likelihood of having had, or intending to have, sex but also with increased likelihood of using contraception [34].

At the general level, studies assessing the quality and quantity of adolescents’ social networks reported little association with sexual health behaviours [51,59]; however, protective effects were noted for some sub-groups of adolescents [54,55] and for other sub-groups social networks were associated with risk taking [54,56]. Three studies reported that adolescents with a peer and/or adult mentor were less likely to engage in sexual risk behaviours [51,55,58] and another reported that peer role models were associated with better outcomes in adolescents from one-parent households [59].

More frequent attendance at religious services was associated with more positive sexual health behaviours [51,55]. However, results in relation to school quality were less consistent, with some elements being associated with better outcomes in some sub-groups of adolescents and presenting as a risk factor in others. There was limited evidence about the role of quality of neighbourhood.

In contrast to the other risk behaviours, there is considerable variation in the role and impact of both FSC and CSC across the indicators of this outcome (e.g. sexual abstinence, sexual experience and contraception use) and across different sub-groups of adolescents. As discussed, it appears that in different contexts different elements of social capital may be more or less salient.

### General risk behaviours

Five studies (see Additional file 2) explored the role and impact of social capital on general health risk behaviours by creating a composite health risk behaviour score that assessed risk across a number of domains (e.g. smoking, alcohol and illicit drug use) [62-66]. Although assessed by five studies, there was limited evidence of the role of FSC in relation to general risk behaviours. Only family structure appeared to have a consistent role to play with children and adolescents, in most instances, benefiting from living in a two-parent household [63-65].

There was limited evidence for social support networks. Two studies assessing peer-based networks reported no association [63,64], but increased contact with neighbours, another indicator of social networks, was associated with fewer risk behaviours [63]. There were mixed findings for civic engagement with some protective [63,65] and some risk relationships identified [62]. Two out of three studies reported that higher quality school environments were associated with fewer risk behaviours [62,63] and another reported that higher quality neighbourhood environments were predictive of better outcomes [65].

### Health risk behaviours – synthesis

Table 1 shows the pattern of impact of the various elements of FSC and CSC on the full range of health risk behaviours. In total, 165 associations between the various health risk behaviours and elements of FSC and CSC were investigated in the 34 included studies: 68 of these associations were positive, showing higher levels of social capital to be associated with better outcomes; 6 were negative, showing higher levels of social capital to be associated with poorer outcomes; and, in 54 cases no association was identified between social capital and the outcome.

**Table 1:** Evidence table showing pattern of investigated associations between social capital and health risk behaviours

**Association**	**Family structure**	**Parent–child relations**	**Adult interest**	**Parental monitoring**	**Extended family support**	**Composite/Other family social capital**	**Social support networks**	**Civic engagement**	**Trust & safety**	**Religiosity**	**Quality of school**	**Quality of neighbourhood**	**Composite/Other community social capital**	**Total**
Number of investigated associations	8	25	2	19	4	9	25	21	4	15	20	4	9	165
Positive	5	10		6		7	4	7	3	10	7	3	6	68
Negative				1			4						1	6
None	1	2	2	11	4	1	10	10		3	9	1		54
Sub-group differences	2	10				1	4	4	1		4		2	28
Inconclusive results		3		1			3			2			0	9

## Discussion

This review provides evidence that social capital is an important construct for understanding young people’s health risk behaviours. Moreover, by delineating the various elements of both FSC and CSC, we have been able to demonstrate that some aspects of social capital are more salient than others in the context of the different behaviours.

We found robust evidence to demonstrate that, across a range of outcomes, social capital generated within the family can be a health asset associated with better health risk behaviour outcomes. Parent–child relationships assessed as being positive were associated with better outcomes and in particular lower levels of tobacco, alcohol and drug use and sexual risk behaviours [42,46,48,50,57]. Moreover, it appears that, in different circumstances, different sub-groups of young people benefited more from this element of social capital than others [34,48,55,59,61]. These findings support those published elsewhere in the literature. For example, there is evidence that parenting styles associated with more positive parent–child relations (e.g. authoritative parenting) are predictive of better educational outcomes in adolescents, and some groups of adolescents appear to benefit more than others [67].

Only when health risk behaviours were assessed using a composite risk score did this pattern fail to replicate; the majority of the associations between the parent–child relationship and general health risk behaviours were neutral. While it was not within the scope of this review to investigate this further, it may be hypothesised that these neutral associations reflect issues of conceptual validity with the aggregation of risk across different behavioural domains. Indeed there is considerable debate within the literature about whether risk behaviours cluster together and, if they do, the ways in which this occurs [5,68-70]. Inappropriate clustering of the risk domains by the individual studies may have resulted in the statistical masking of positive and/or negative associations.

In the synthesis of evidence across the outcomes, the majority of the associations with parental monitoring were neutral and this might lead to the conclusion that parental monitoring, or parental control, is of little value in the context of health risk behaviours. Indeed, this appears to be case for tobacco, alcohol and drug use and general health risk behaviours. However, parental monitoring was associated with better sexual health outcomes. This different pattern may be explained, in part, by the fact that sexual activity is different to other risk behaviours where abstinence is the preferred outcome (e.g. tobacco use). Sex is a natural part of development for most adolescents and at the various stages of adolescence it may be considered more or less appropriate, by both the adolescent and others around them. This sense of appropriateness will necessarily interact with support structures, including those within the family, that are designed to prevent younger adolescents from engaging in sex and promote safer sex amongst older adolescents. Indeed, some of the studies in this review reported that parental monitoring was only related to reduced sexual risk taking in younger age groups [56]. The identification of a different pattern of association between social capital and health risk behaviours has implications for intervention development and delivery in that a single intervention may not be effective in reducing risk across different outcomes simultaneously [71].

The evidence for the role of family structure was limited but suggests that, while young people benefit from living with at least one biological parent, being in a two-parent family was most protective [50,57,63-65]. A limited number of studies assessed the role of adult interest in the child, and extended family support, but there was no evidence to suggest that these elements of FSC had a negative impact on health risk outcomes.

As with FSC, there were elements of CSC that were more or less salient across the various health risk behaviours. However, CSC also showed evidence of the ‘downside’ of social capital with some associations linking it to increased likelihood of young people engaging in health risk behaviours. Specifically, young people with indicators of wider peer networks were at increased risk of using tobacco [41,42]. We also found inconsistent patterns of association between social support networks and alcohol, drug use, sexual health and general risk behaviours, with some studies reporting social networks as health assets and others finding them to be health liabilities. Others have previously reported the ‘downside’ of social capital [72-74] and, in the context of young people, we might hypothesise that while some social networks may support young people in some circumstances (e.g. in the development of social competencies) they may also create opportunity and encouragement to experiment with behaviours that might be considered (by adults) as undesirable for this age group, including health risk behaviours. Thus it essential that future research endeavours to disentangle and characterise the various types of social networks that young people are embedded in and identifies those more likely to support positive health outcomes.

Linked to this, our search did not identify any studies that explored the role of social relationships and networks sustained in the online world and health risk behaviour outcomes. This is surprising given that online social networking (e.g. *Facebook*) is recognised as being increasingly important for children and adolescents [75] and evidence that relationships formed and sustained online are an important source of social capital, with the potential to influence psychological wellbeing, for young people in their late teens and early twenties [76]. The lack of evidence about social capital generated online perhaps reinforces the need, as noted above, for definitions of social capital to be reconceptualised to capture the different social spaces and networks of young people [21,25].

In other CSC domains, this review found limited and inconsistent evidence about the role and impact of civic engagement and the quality of the neighbourhood the young people live in. On the other hand, high quality school environments were associated with reduced tobacco, alcohol and drug use and lower levels of general health risk behaviours; only in the context of sexual health behaviours was the role of school less clear. Neighbourhoods have been identified as important social capital arena for adults but, although there is evidence that lower quality neighbourhoods are associated with poorer outcomes for young people (e.g. child maltreatment) [77], it is acknowledged that young people navigate and interact with their local communities differently to adults [27] and this is likely to result in neighbourhood quality having a different role to play in the health and wellbeing of youth [25]. On the other hand, school is where young people spend a significant amount of their time and schools that are high in social capital and/or support young people in creating and mobilising their own social capital evidently support young people to make better choices about behaviours that may carry health risks. Acknowledging the importance of the school environment in helping to develop happy, confident individuals who will do well in life, UK curricula encourage schools to address issues relevant to wellbeing, including making healthy choices in relation to tobacco, alcohol, illicit drugs and sexual health [78,79].

Finally, religiosity was identified as an asset related to better tobacco, alcohol, drug and sexual health outcomes [33,36,44,46,47,55,59,63]. General health risk was the only domain where this was not consistently replicated and this may be related to the issues of conceptual validity noted above. It is important to note, however, that only social elements of religiosity (e.g. frequency of service attendance) were associated with health outcomes; there was no evidence that the personal importance of religion was linked with health risk behaviours. It may be hypothesised that the social elements of religiosity are illustrative of the young person’s access to bonding social capital through faith-based support networks. These networks might exert influence over the young person’s health risk behaviours through expectations in relation to the behavioural norms of the group [80].

### Strengths and limitations

As with any review, the results need to be considered in relation to the strengths and weaknesses both of the review process itself and the included studies. In relation to the process, a major strength of this review lies in the integrative approach which enabled the capture and synthesis of a large body of evidence generated from across different study designs. That said we are cognisant that, as with any review, relevant literature may not have been captured. For example, publication bias, the tendency for journals to publish studies reporting statistically significant results, may have limited our access to studies that did not find a relationship between social capital and health risk behaviour outcomes. In addition, we were constrained by the adequacy of the indexing within each of the databases and, while we placed no geographic restrictions on the selection of studies, employing an English language-only criterion may have resulted in the exclusion of relevant studies published in other languages. We did, however, employ a number of strategies to broaden the reach of our search, including hand searching relevant references lists and web-sites.

In terms of the individual studies, while the synthesised results offer a strong body of evidence demonstrating the association between FSC and CSC and health risk behaviour outcomes, the majority of the included studies were cross-sectional surveys. This design limits our ability to make inferences about the direction of causation in these associations and the mechanisms of action. It might be hypothesised, for example, that social capital acquired in the context of family and community is an asset that helps protect young people from engaging in health risk behaviours [21,37]. Conversely, it could be argued that experimenting with behaviours that transgress the boundaries of social acceptability (at least in the eyes of adults), young people are more likely to report negative relationships within and between their family and community. In order to develop social capital interventions that effect better health outcomes, further evidence that demonstrates both the mechanisms that link social capital to health and the direction of causation is needed.

There was considerable heterogeneity in the definition and measurement of social capital across the studies. While this made synthesising the available evidence challenging, all data were extracted using well defined study-specific inclusion/exclusion criteria and by a minimum of two reviewers. There was also heterogeneity in the individuals asked to report on social capital; data were obtained variously from the young people themselves and adults, such as their parents. While it was not possible within the remit of this review to give full consideration to the impact of the different reporters, it is important to be mindful of this. Previous research exploring the relationship between parenting style and adolescent outcome variables has demonstrated that there can be considerable divergence in the reports of parenting style offered by young people and their parents and these different reports share different relationships with the outcome variables [81].

Finally, in terms of the individual studies, although the purpose of this review was to consider the role and impact of social capital on the health risk behaviour outcomes of young people, all accept one of the studies had adolescent-only samples and the focus was very much on mid- to late-adolescence. Given the nature of the behavioural outcomes, the lack of evidence about pre- and early adolescent years is perhaps understandable; however, it limits our capacity to draw conclusions about these age groups.

## Conclusions

This is, to the best of our knowledge, the first systematic review to synthesise empirical evidence linking social capital to a range of health risk behaviour outcomes and our findings have important implications for future policy, practice and research. Health risk behaviour interventions targeted at young people have traditionally failed to take account of the importance of social capital but this review demonstrates that both family and community social capital have an important role to play. Moreover, by looking within and across the various health risk behaviours we have been able to identify and highlight the elements of social capital that present as supportive health assets irrespective of the behavioural domain (e.g. positive parent–child relations) and highlight elements of social capital that may in some instances present as a health liability (e.g. peer-based social support networks) and thus need careful consideration in the context of intervention development.

International policy emphasises the need for early intervention as a means of reducing inequalities and promoting health and wellbeing across the lifespan, and priorities for Europe include the promotion of resilient families and communities [82]. Our findings illustrate ways in which family and community social capital might be generated and exploited to promote resilience and, ultimately, better health outcomes.

## Competing interests

KEM, SK, AM, EM and FC were commissioned to undertake this work by the Glasgow Centre for Population Health. JM and JE commissioned the work on behalf of the Glasgow Centre for Population Health.

## Authors’ contributions

All authors were involved in developing the scope of the project and in determining the search strategy. The search was conducted by KEM. The study selection process, data extraction and quality appraisal was undertaken by KEM, SK, EM and FC. Data analysis and synthesis was conducted by KEM. All authors contribution to the drafting of the manuscript and read and approved the final version of the manuscript.

## Pre-publication history

The pre-publication history for this paper can be accessed here:

http://www.biomedcentral.com/1471-2458/13/971/prepub

## Supplementary Material

Additional file 1 Search strategy (PsycINFO). Click here for file

Additional file 2 Description of studies included in the review (ordered by outcome). Click here for file
