# Supplementary material for: The association between family and community social capital and health risk behaviours in young people: an integrative review
Source: BMC Public Health. 2013 Oct 19;13:971. doi: 10.1186/1471-2458-13-971 (PMC4015354; doi:10.1186/1471-2458-13-971)
Supplement: Additional file 1 — Search strategy (PsycINFO). [file 1471-2458-13-971-S1.docx]

# Additional File 1: Search strategy (PsycINFO)

| **Topic** | **Index terms and keywords** |
| --- | --- |
| **Social capital** | S1 social networks/ OR online social networks/  S2 social support/  S3 friendship/  S4 friend*  S5 peer relations/ OR peer pressure/  S6 family relations/ OR child discipline/ OR childrearing practices/ OR family conflict/ OR marital relations/ OR parent child relations/ OR parental role/ OR sibling relations  S7 intergenerational relations/  S8 parenting/ OR authoritarian parenting/ OR childrearing practices/ OR parent child communication/ OR parental involvement/ OR parenting style/ OR permissive parenting/  S9 school environment/  S10 school*  S11 community involvement/  S12 neighborhoods/ OR ghettoes/  S13 social structure/  S14 social capital/  S15 social capital  S16 salutogenesis OR asset* based OR health asset*  S17 trust (social behaviour)/ OR reciprocity/  S18 trust OR reciprocity  S19 social cohesion OR neighbourhood cohesion  S20 family social capital OR family capital  S21 community social capital OR community capital  S22 community processes/  S23 OR/1-22 |
| **Psycho-social health and wellbeing** | S24 mental health/ OR community mental health/  S25 emotional intelligence/  S26 psychological development/  S27 cognitive development/ OR intellectual development/ OR language development/ OR language delay  S28 emotional development/  S29 psychosocial development/ OR childhood play/ OR psychosexual development/  S30 health behavior/  S31 well being/  S32 quality of life/  S33 emotional adjustment/ OR emotional control/ OR identity crisis/  S34 public health/  S35 health promotion/  S36 behavior problems/  S37 child psychology/ OR adolescent psychology/  S38 adolescent development/  S39 childhood development/ OR early childhood development/ OR proximal development  S40 OR/24-39 |
| **Combined search** | S41 S23 AND S40 |
| **Combined search with delimiters** | S42 limit S41 as follows:  publication year – 1990-2012  peer reviewed journal articles  English language  age groups – neonatal (birth-1 month), infancy (2-23 months), preschool (2-5 years), school age (6-12 years), adolescence (13-17 years)  population group – humans |
