# Supplementary material for: The association between family and community social capital and health risk behaviours in young people: an integrative review
Source: BMC Public Health. 2013 Oct 19;13:971. doi: 10.1186/1471-2458-13-971 (PMC4015354; doi:10.1186/1471-2458-13-971)
Supplement: Additional file 2 — Description of studies included in the review (ordered by outcome). [file 1471-2458-13-971-S2.doc]

## Additional file 2 Description of studies included in the review (ordered by outcome)

| **Authors, Country**  **Quality rating** | **Design** | **Sample** | **Assessed**  **FSC CSC** | | **Outcomes** |
| --- | --- | --- | --- | --- | --- |
| **1) Tobacco use** | | | | | |
| Atkins et al 2002 [33]  USA  high quality | cross-sectional survey | size: 1,256  age group: adolescents  age range: 13-19yrs  sex: 52% female  ethnic group: 48% White | ✓ | ✓ | Positive parent-adolescent communication, recreational group membership, active citizenship, attendance at religious services/groups and having a peer/adult role model were all associated with increased odds of non-use of tobacco. |
| Aim: To examine the influence of individual developmental assets on youth tobacco use. | | | | | |
| Borawski et al 2003 [34]  USA  high quality | cross sectional survey | size: 692  age group: adolescents  mean age: 15.7 yrs  sex: 50% female  ethnic group: 41% White | ✓ | 🗶 | Female, but not male, adolescents reporting that their relationship with their parents is high in trust were more likely to not use tobacco. Parental monitoring and negotiated unsupervised time with peers had no role in relation to tobacco use. |
| Aim: To explore parental monitoring, negotiated unsupervised time and trust as they relate to tobacco use. | | | | | |
| Fulkerson et al 2006 [35]  USA  high quality | cross sectional survey  data collected 1996-97 | size: 98,340  age group: adolescents  age range: 11-17 yrs  sex: 50% female  ethnic group: 86% White | ✓ | 🗶 | Adolescents from families who ate together more frequently (an indicator of family cohesion) were less likely to report tobacco use. |
| Aim: To examine the associations between family meal frequency, developmental assets and tobacco use among a national sample of adolescents. | | | | | |

| **Authors, Country**  **Quality rating** | **Design** | **Sample** | **Assessed**  **FSC CSC** | | **Outcomes** |
| --- | --- | --- | --- | --- | --- |
| Mellor et al 2011 [36]  USA  low quality | cross-sectional survey (ADD Health)  data collected 1995 | size: 10.972  age group: adolescents  age range: 13-18 yrs  sex: not stated  ethnic group: not stated | 🗶 | ✓ | After controlling for other variables results were inconsistent for the impact of religious participation on smoking. |
| Aim: To identify the effects of religious participation on smoking. | | | | | |
| Morgan et al 2009 [37]  UK  moderate quality | cross-sectional survey (Health Behaviour in School-aged Children) | size: 6,425  age group: adolescents  age range: 11-15 yrs  sex: 51% female  ethnic group: not stated | ✓ | ✓ | Low family sense of belonging and lower quality of neighbourhood associated with more frequent smoking. No role for parental monitoring, social support networks, civic engagement or quality of school. |
| Aim: To assess the importance of sense of belonging, autonomy, control and social networking on a range of health-related outcomes (including smoking). | | | | | |
| Smith et al 2009 [38]  USA  low quality | cross sectional survey | size: 61  age group: adolescents  age range: 11-13 yrs  sex: 51% female  ethnic group: 53% Black | ✓ | ✓ | Neither family nor community social capital associated with tobacco use. |
| Aim: To identify assets that contribute to positive youth outcomes, specifically not engaging in risk behaviour (including smoking). | | | | | |
| Takakura 2011 [39]  Japan  high quality | cross-sectional survey | size: 2,424  age group: adolescents  age range: 15-18 yrs  sex: 52% female  ethnic group: not stated | 🗶 | ✓ | Low individual-level trust associated with increased odds of smoking in adolescents. Low school-level trust associated with increased odds of smoking in females only. |
| Aim: To examine the individual and contextual effects of social capital at school on cigarette smoking. | | | | | |

| **Authors, Country**  **Quality rating** | **Design** | **Sample** | **Assessed**  **FSC CSC** | | **Outcomes** |
| --- | --- | --- | --- | --- | --- |
| Vuille et al 2002 [40]  Switzerland  high quality | cross-sectional survey | size: 459  age group: adolescents  mean range: 14.8 yrs  sex: 54% female  ethnic group: 55% Swiss | 🗶 | ✓ | Positive school climate associated with reduced prevalence of smoking. |
| Aim: To look into details of the relationships between smoking, individual psychological variables, and school climate. | | | | | |
| Wen et al 2009 [41]  USA  high quality | longitudinal survey (ADD Health)  data collected 1994-96 | size: 13,552  age group: adolescents  age range: not stated  sex: 51% female  ethnic group: 54% White | ✓ | ✓ | Parent-adolescent relations a risk factor, diluted by protective effect of a close relationship. Parental monitoring protective against smoking. More frequent contact with friends a risk factor. No role for family structure, quality of school or neighbourhood. |
| Aim: To investigate factors influencing adolescent cigarette smoking at the individual-, peer-, school-, and state-level. | | | | | |
| Yugo et al 2007 [42]  Canada  low quality | cross-sectional survey (National Longitudinal Survey of Children and Youth)  data collected 2000-01 | size: 3,725  age group: adolescents  age range: 12-15 yrs  sex: not stated  ethnic group: not stated | ✓ | ✓ | Higher parental nurturance and school engagement associated with reduced odds of tobacco use. Higher peer connectedness associated with increased odds of tobacco use. No role for parental monitoring or civic engagement. |
| Aim: To examine which assets account for the most variance in participation in risky health behaviours (including smoking). | | | | | |
| Zambon et al 2010 [43]  Belgium, Canada, England, Italy, Poland, Romania  high quality | cross sectional survey (Health Behaviour in School-aged Children)  data collected 2005-06 | size: 10,230  age group: adolescents  age range: 15 yrs  sex: 53% females  ethnic group: not stated | 🗶 | ✓ | In general, being a member of at least one recreational club was protective against smoking. Differential impact found across individual club types. |
| Aim: To test whether young people’s participation in clubs is associated with better health and healthier behaviours (including not smoking). | | | | | |

| **Authors, Country**  **Quality rating** | **Design** | **Sample** | **Assessed**  **FSC CSC** | | **Outcomes** |
| --- | --- | --- | --- | --- | --- |
| **2) Alcohol consumption** | | | | | |
| Bartkowski et al 2007 [44]  USA  high quality | cross-sectional survey (Monitoring the Future) | size: 1,630  age group: adolescents  age range: not stated  sex: not stated  ethnic group: not stated | 🗶 | ✓ | Secular (but not religious) civic participation, humanistic (but not religious) trust, and attendance at religious services all associated with reduced alcohol use. Inconsistent findings for denominational affiliation and religious saliency. |
| Aim: To adopt a social capital perspective on religiosity and adolescent alcohol use. | | | | | |
| Borawski et al 2003 [34]  USA  high quality | cross-sectional survey | size: 692  age group: adolescents  mean age: 15.7 yrs  sex: 50% female  ethnic group: 41% White | ✓ | 🗶 | Negotiated unsupervised time with peers associated with increased odds of reporting of alcohol use in adolescents. Parental monitoring and trust were associated with reduced odds of reporting of alcohol use in males only. |
| Aim: To explore parental monitoring, negotiated unsupervised time and trust as they relate to alcohol use. | | | | | |
| Eitle et al 2009 [45]  USA  moderate quality | longitudinal survey (ADD Health)  data collected 1994+ | size: 7,637  age group: adolescents  age range: 13-18 yrs  sex: not stated  ethnic group: 80% White | ✓ | ✓ | Living in a two-parent household was protective in relation to alcohol use and binge drinking for Cuban and Mexican, but was a risk for Puerto Rican, adolescents. Positive parent-child relations protective for Mexican adolescents only. Extended family support protective for Mexican and Puerto Rican, but a risk factor for Cuban, adolescents. Increased frequency of parental communication with adolescents’ friends’ parents was only protective for Mexican adolescents. |
| Aim: To examine associations among immigrant generation, selective acculturation, and alcohol use in Mexican, Cuban, and Puerto Rican adolescents. | | | | | |

| **Authors, Country**  **Quality rating** | **Design** | **Sample** | **Assessed**  **FSC CSC** | | **Outcomes** |
| --- | --- | --- | --- | --- | --- |
| Fulkerson et al 2006 [35]  USA  high quality | cross-sectional survey | size: 98,340  age group: adolescents  age range: 11-17 yrs  sex: 50% female  ethnic group: 86% White | ✓ | 🗶 | Adolescents reporting that their family ate together more frequently (an indicator of family cohesion) had reduced odds of using alcohol. |
| Aim: To examine the associations between family meal frequency, developmental assets and high-risk behaviours (including alcohol consumption) in adolescents. | | | | | |
| Mellor et al 2011 [36]  USA  low quality | cross-sectional survey (ADD Health) | size: 10,972  age group: adolescents  age range: 13-18 yrs  sex: not stated  ethnic group: not stated | 🗶 | ✓ | After controlling for other variables results were inconsistent for the impact of religious participation on binge drinking. |
| Aim: To identify the effects of religious participation on binge drinking. | | | | | |
| Morgan et al 2009 [37]  UK  moderate quality | cross-sectional survey (Health Behaviour in School-aged Children) | size: 6,425  age group: adolescents  age range: 11-15 yrs  sex: 51% female  ethnic group: not stated | ✓ | ✓ | Perceptions of high father control behaviour, low levels of joint family activity, limited opportunity for decision making at school and low sense of belonging to school were associated with increased odds of regular drinking. No role for social support networks. |
| Aim: To assess importance of sense of belonging, autonomy, control & social networking on a range of health-related outcomes (including alcohol consumption). | | | | | |
| Oman et al 2004 [46]  USA  high quality | cross-sectional survey | size: 1,255  age group: adolescents  age range: 13-19 yrs  sex: 52% female  ethnic group: 48% White | ✓ | ✓ | Positive parent-adolescent communication, having a peer role model and more frequent religious services attendance associated with non-use of alcohol. Active citizenship protective only for adolescents from 1-parent households. |
| Aim: To examine the relationship between youth assets and alcohol use in a community sample. | | | | | |

| **Authors, Country**  **Quality rating** | **Design** | **Sample** | **Assessed**  **FSC CSC** | | **Outcomes** |
| --- | --- | --- | --- | --- | --- |
| Rasic et al 2011 [47]  Canada  moderate quality | cross-sectional survey | size: 1,615  age group: adolescents  age range: 15-19 yrs  sex: 49% females  ethnic group: not stated | 🗶 | ✓ | Less frequent religious service attendance associated with increased odds of binge drinking. No role for personal importance of religion. |
| Aim: To examine the impact of social capital on the relationships between religion and substance use disorders. | | | | | |
| Smith et al 2009 [38]  USA  low quality | cross-sectional survey | size: 61  age group: adolescents  age range: 11-13 yrs  sex: 51 female  ethnic group: 53% Black | ✓ | ✓ | No association identified between any element of family or community social capital and alcohol use. |
| Aim: To identify assets that contribute to positive youth outcomes specifically health behaviours and not engaging in risk behaviour (including alcohol use). | | | | | |
| Springer et al 2006 [48]  El Salvador  high quality | cross-sectional survey | size: 930  age group: adolescents  age range: 12-19 yrs  sex: 48%  ethnic group: not stated | ✓ | ✓ | Poorer parent-adolescent relationships associated with increased odds of binge drinking in female adolescents. Low levels of school cohesion associated with increased odds of binge drinking in females but reduced odds of binge drinking in males. |
| Aim: To examine perceived parental social support and social cohesion at school with the prevalence of a range of youth health risk behaviours (including alcohol use). | | | | | |
| Takakura 2011 [39]  Japan  high quality | cross-sectional survey | size: 2,424  age group: adolescents  age range: 15-18 yrs  sex: 52% female  ethnic group: not stated | 🗶 | ✓ | Low individual trust was associated with increased odds of drinking in females only. No role identified for school-level trust. |
| Aim: To examine the individual and contextual effects of social capital at school on alcohol drinking. | | | | | |

| **Authors, Country**  **Quality rating** | **Design** | **Sample** | **Assessed**  **FSC CSC** | | **Outcomes** |
| --- | --- | --- | --- | --- | --- |
| Windle 1994 [49]  USA  high quality | longitudinal cohort study | size: 1,098  age group: adolescents  mean age: 16.2 yrs  sex: 52% female  ethnic group: 98% White | 🗶 | ✓ | Poorer quality friendships (e.g. high in hostility) associated with higher alcohol consumption. This was significant in cross-sectional, but not longitudinal, analyses. |
| Aim: To study concurrent and prospective relations between friendship characteristics and adolescent externalising & internalising problems (including alcohol consumption). | | | | | |
| Yugo et al 2007 [42]  Canada  low quality | cross-sectional survey (Longitudinal Survey of Children & Youth)  data collected 2000-01 | size: 3,725  age group: adolescents  age range: 12-15 yrs  sex: not stated  ethnic group: not stated | ✓ | ✓ | Higher parental nurturance and school engagement associated with reduced odds of using alcohol. Higher peer connectedness associated with increased odds of alcohol use. No role for parental monitoring or civic engagement. |
| Aim: To examine which assets account for the most variance in positive health outcomes and participation in risky health behaviours (including alcohol use). | | | | | |
| Zambon et al 2010 [43]  Belgium, Canada, England, Italy, Poland, Romania  high quality | cross-sectional survey (Health Behaviour in School-aged Children)  data collected 2005-06 | size: 10,230  age group: adolescents  age range: 15 yrs  sex: 53% female  ethnic group: not stated | 🗶 | ✓ | In general, being a member of at least one recreational club was protective against drunkenness. Differential impact found across individual club types. |
| Aim: To test whether young people’s participation in clubs is associated with better health and healthier behaviours (including less drunkenness). | | | | | |
| **3) Drug use** | | | | | |
| Bartkowski et al 2007 [44]  USA  high quality | cross-sectional survey (Monitoring the Future) | size: 1,630  age group: adolescents  age range: not stated  sex: not stated  ethnic group: not stated | 🗶 | ✓ | Trust in people (but not trust in God) and attendance at religious services associated with reduced marijuana and other drug use. Secular (not religious) civic participation and personal importance of religion associated with reduced marijuana use but not other drugs. |
| Aim: To adopt a social capital perspective on religiosity and adolescent drug use. | | | | | |

| **Authors, Country**  **Quality rating** | **Design** | **Sample** | **Assessed**  **FSC CSC** | | **Outcomes** |
| --- | --- | --- | --- | --- | --- |
| Borawski et al 2003 [34]  USA  high quality | cross-sectional survey | size: 692  age group: adolescents  mean age: 15.7 yrs  sex: 50% female  ethnic group: 41% White | ✓ | 🗶 | High trust adolescent-parent relationships associated with decreased odds of marijuana use in females only. Negotiated unsupervised time with peers associated with increased odds of marijuana use. No role for parental monitoring. |
| Aim: To explore parental monitoring, negotiated unsupervised time and trust as they relate to drug use. | | | | | |
| Fulkerson et al 2006 [35]  USA  high quality | cross-sectional survey | size: 98,340  age group: adolescents  age range: 11-17 yrs  sex: 50% female  ethnic group: 86% White | ✓ | 🗶 | Adolescents from families who ate together more frequently (an indicator of family cohesion) were less likely to report using illicit drugs. |
| Aim: To examine the associations between family meal frequency, developmental assets and high-risk behaviours (including drug use) among adolescents. | | | | | |
| Mellor et al 2011 [36]  USA  low quality | cross-sectional survey (ADD Health) | size: 10,972  age group: adolescents  age range: 13-18 yrs  sex: not stated  ethnic group: not stated | 🗶 | ✓ | Adolescents who attended religious services more frequently were less likely to report marijuana use. |
| Aim: To identify the effects of religious participation on marijuana use. | | | | | |
| Oman et al 2004 [46]  USA  high quality | cross-sectional survey | size: 1,255  age group: adolescents  age range: 13-19 yrs  sex: 52% female  ethnic group: 48% White | ✓ | ✓ | More positive parent-adolescent relationships, participation in recreational clubs and religious services, civic engagement and having a peer/adult role model all associated with increased odds of never having used drugs. |
| Aim: To examine the relationship between youth assets and drug use in a community sample. | | | | | |

| **Authors, Country**  **Quality rating** | **Design** | **Sample** | **Assessed**  **FSC CSC** | | **Outcomes** |
| --- | --- | --- | --- | --- | --- |
| Rasic et al 2011 [47]  Canada  moderate quality | cross-sectional survey | size: 1,615  age group: adolescents  age range: 15-19 yrs  sex: 49% females  ethnic group: not stated | 🗶 | ✓ | In females, low attendance at religious services, but not religious importance, associated with increased marijuana use. In males, low importance of religion, but not religious attendance, associated with increased marijuana use. |
| Aim: To examine impact of social capital on the relationships between religion and substance use disorders (including drug use). | | | | | |
| Smith et al 2009 [38]  USA  low quality | cross-sectional survey | size: 61  age group: adolescents  age range: 11-13 yrs  sex: 51 female  ethnic group: 53% Black | ✓ | ✓ | No association identified between any element of family or community social capital and drug use. |
| Aim: To identify assets that contribute to positive youth outcomes, specifically health behaviours and not engaging in risk behaviour (including drug use). | | | | | |
| Springer et al 2006 [48]  El Salvador  high quality | cross-sectional survey | size: 930  age group: adolescents  age range: 12-19 yrs  sex: 48%  ethnic group: not stated | ✓ | ✓ | Low perceived parental support associated with increased odds of reporting drug use. Low cohesion at school associated with increased odds of drug use in females. |
| Aim: To examine perceived parental social support and perceived social cohesion at school with the prevalence of a range of youth health risk behaviours (including drug use). | | | | | |
| Yugo et al 2007 [42]  Canada  low quality | cross-sectional survey (National Longitudinal Survey of Children & Youth)  data collected 2000-01 | size: 3,725  age group: adolescents  age range: 12-15 yrs  sex: not stated  ethnic group: not stated | ✓ | ✓ | Higher parental nurturance and school engagement associated with reduced odds of using marijuana. Higher peer connectedness associated with increased odds of marijuana use. No role for parental monitoring or civic engagement. |
| Aim: To examine which assets account for the most variance in positive health outcomes and participation in risky health behaviours (including drug use). | | | | | |

| **Authors, Country**  **Quality rating** | **Design** | **Sample** | **Assessed**  **FSC CSC** | | **Outcomes** |
| --- | --- | --- | --- | --- | --- |
| **4) Sexual health** | | | | | |
| Bensyl et al 2011 [51]  USA  moderate quality | cross-sectional survey | size: 2,335  age group: adolescents age:  sex range: 12-19 yrs  ethnic group: 44% White | ✓ | ✓ | Frequent attendance at religious services associated with increased odds of never having had sex, apart from those from most impoverished neighbourhoods. Having a role model was protective for all adolescents. No role for extended family support, social support or civic engagement. |
| Aim: To explore the associations among household income, youth assets, and youth sexual intercourse. | | | | | |
| Borawski et al 2003 [34]  USA  high quality | cross-sectional survey | size: 692  age group: adolescents  mean age: 15.7 yrs  sex: 50% female  ethnic group: 41% White | ✓ | 🗶 | High trust adolescent-parent relationships associated with lower intention to have sex in females only. Parental monitoring associated with consistent condom use in males only. Negotiated unsupervised time with peers associated with having had/intention to have sex and contraception use. |
| Aim: To explore parental monitoring, negotiated unsupervised time and trust as they relate to sexual activity. | | | | | |
| Crosby et al 2003 [52]  USA  high quality | cross-sectional survey (Youth Risk Behaviour Surveillance Survey) | size: 15,349  age group: adolescents  age range: 14-18 yrs  sex: 50% female  ethnic group: 61% White | 🗶 | ✓ | State-level social capital associated with better sexual health. |
| Aim: To assess the state-level relationship between Putnam’s index of social capital and sexual risk and protective behaviours of adolescents in 28 U.S. states. | | | | | |

| **Authors, Country**  **Quality rating** | **Design** | **Sample** | **Assessed**  **FSC CSC** | | **Outcomes** |
| --- | --- | --- | --- | --- | --- |
| Erulkar et al 2009 [53]  Ethiopia  moderate quality | cross-sectional survey | size: 521  age group: adolescents age range: 15-19 yrs  sex: 100% female  ethnic group: not stated | 🗶 | ✓ | Lack of social support networks predicted non-consensual sexual debut but not early sexual initiation. |
| Aim: To explore the role of social exclusion in sexual debut and non-consensual sexual debut. | | | | | |
| Evans et al 2004 [54]  USA  high quality | cross-sectional survey | size: 2,108  age group: adolescents age range: 14-18 yrs  sex: 44% female  ethnic group: 61% Black | ✓ | ✓ | Increased adult support associated with low sexual risk taking in White females and Black males but more sexual risk taking in White males. No role for other social capital variables. |
| Aim: To explore which youth assets were predictive of youth engagement in risky sexual behaviours. | | | | | |
| Fulkerson et al 2006 [35]  USA  high quality | cross-sectional survey | size: 98,340  age group: adolescents  age range: 11-17 yrs  sex: 50% female  ethnic group: 86% White | ✓ | 🗶 | Adolescents from families who ate together more frequently (an indicator of family cohesion) were less likely to report having had sexual intercourse. |
| Aim: To examine the associations between family meal frequency, developmental assets and high-risk behaviours (including sexual intercourse) among adolescents. | | | | | |
| Harris et al 2006 [55]  USA  high quality | cross-sectional survey | size: 1,079  age group: adolescents  age range: 13-19 yrs  sex: 52% female  ethnic group: 49% White | ✓ | 🗶 | Family and community social capital associated with never having had sex in adolescents supervised full-time at home. Having a peer role model and increased religious service attendance were protective for adolescents home alone for 2 hours or more per day. |
| Aim: To examine how relationships between youth assets and sexual intercourse vary according to the stratification of youth ‘self-care’ and ‘supervised’ at home youth. | | | | | |

| **Authors, Country**  **Quality rating** | **Design** | **Sample** | **Assessed**  **FSC CSC** | | **Outcomes** |
| --- | --- | --- | --- | --- | --- |
| Hellerstedt et al 2006 [56]  USA  high quality | cross-sectional survey (Minnesota Student Survey)  data collected 1998 & 2001 | size: 4,135  age group: adolescents  age range: 13-18 yrs  sex: 100% female  ethnic group: American Indian | ✓ | ✓ | Living with father associated with reduced odds of being sexual experienced in younger, but not older, adolescents. Participation in sport clubs was a risk factor for older males. Positive relationship with teacher was associated with increased odds of being sexually experienced in younger females and reduced odds in older males. Other school quality variables were protective for all groups. |
| Aim: To examine the correlates of having ever had sexual intercourse among American Indians in Minnesota. | | | | | |
| Kerrigan et al 2006 [57]  USA  high quality | cross-sectional survey (Perceived Risk of Sexually Transmitted Disease Survey) | size: 343  age group: adolescents  age range: 14-19 years  sex: 76% female  ethnic group: 98% Black | ✓ | ✓ | Living with at least 1 biological parent, positive communication with parents and living in a cohesive neighbourhood associated with increased odds of condom use. No role for parental or neighbourhood monitoring. |
| Aim: To examine the associations between perceived neighbourhood social cohesion and collective monitoring and condom use. | | | | | |
| Oman et al 2004 [58]  USA  moderate quality | cross-sectional survey | size: 1,121  age group: adolescents  age range: 13-19 yrs  sex: 53% female  ethnic group: 49% White | ✓ | ✓ | Family and community assets protective for some of the sexual health outcomes. The more assets the adolescent had the more protection they gained for some of the sexual health outcomes. No association between assets and current sexual activity or number of sexual partners. |
| Aim: To investigate the possible positive cumulative effects that youth assets may have on youth sexual behaviour**.** | | | | | |
| Oman et al 2005 [59]  USA  high quality | cross-sectional survey | size: 1,253  age group: adolescents  age range: 13-19 yrs  sex: 52% female  ethnic group: 49% White | ✓ | ✓ | Family communications, active citizenship, religious participation and having peer/adult role models were protective for a number of sexual health outcomes. The importance of some assets differed across youth from 1- or -2-parent households. No role for social support networks. |
| Aim: To investigate how the relationship between youth assets and sexual risk behaviours vary according to family structure. | | | | | |
| **Authors, Country**  **Quality rating** | **Design** | **Sample** | **Assessed**  **FSC CSC** | | **Outcomes** |
| Parkes et al 2011 [60]  UK  high quality | cross-sectional survey | size: 1,854  age group: adolescents  mean age: 16.6 yrs  sex: 50% female  ethnic group: 94% White | ✓ | 🗶 | Parental supportiveness, ease of communication and parental monitoring associated with better sexual health outcomes. Frequency of communication with parents about sex associated with not delaying first sex. |
| Aim: To identify processes associated with sexual risk avoidance, autonomy and relatedness. | | | | | |

| Springer et al 2006 [48]  El Salvador  high quality | cross-sectional survey  data collected 1999 | size: 930  age group: adolescents  age range: 12-19 yrs  sex: 48%  ethnic group: not stated | ✓ | ✓ | Positive support from parents associated with higher odds of sexual abstinence in females. No role for quality of school. |
| --- | --- | --- | --- | --- | --- |
| Aim: To examine perceived parental social support and perceived social cohesion at school with the prevalence of a range of youth health risk behaviours (including sex). | | | | | |
| Tolma et al 2011 [61]  USA  high quality | cross-sectional survey (Youth Assets Survey)  data collected 2003-04 | size: 976  age group: adolescents  age range: 12-19 years  sex: 53% female  ethnic group: 43% White | ✓ | 🗶 | Increased parental monitoring associated with increased odds of sexual abstinence. Elements of the parent-adolescent relationship were protective for Black and White, but not Hispanic, adolescents. |
| Aim: To examine the relationship between parental youth assets and race/ethnicity and sexual abstinence. | | | | | |
| Wight et al 2006 [50]  UK  high quality | longitudinal survey  data collected in 1996 & 1999  Time 1 (T1), Time 2 (T2) | size: 5,041  age group: adolescents  age range T1: 13-14 yrs  age range T2: 15-16 yrs  sex: 54% female  ethnic group: not stated | ✓ | 🗶 | Living away from father associated with future risk behaviour in males. Living in a 1-parent house associated with future risk behaviour in females. Feeling at ease talking to parents about sex protective against early first sex for females. Increased parental monitoring was protective for both sexes but girls benefited the most. |
| Aim: To use longitudinal data to show how parental monitoring predicts sexual behaviour. | | | | | |

| **Authors, Country**  **Quality rating** | **Design** | **Sample** | **Assessed**  **FSC CSC** | | **Outcomes** |
| --- | --- | --- | --- | --- | --- |
| **5) General risk behaviours** | | | | | |
| Jager et al 2011 [66]  USA  moderate quality | longitudinal survey (ADD Health) | size: 4,233  age group: adolescents  age range: 13-19 yrs  sex: not stated  ethnic group: not stated | ✓ | ✓ | Different patterns of positive relationships with others share different relationships with problem behaviours. |
| Aim: To examine heterogeneity in adolescent relationship constellations and its relation to adolescent binge drinking, marijuana use and sexual health. | | | | | |
| Reininger et al 2005 [62]  USA  high quality | cross-sectional survey | size: 3,439  age group: adolescents  age range: 14-18 yrs  sex: 53% female  ethnic group: 50% Black | ✓ | ✓ | Higher perceived support from school associated with lower risk scores. No role for other community social capital indicators. No role for family social capital. |
| Aim: To examine the relationship between smoking, drinking and sex, assets, and demographic characteristics. | | | | | |
| Smylie et al 2006 [63]  Canada  high quality | cross-sectional survey (National Population Health Survey) | size: 4,178  age group: adolescents  age range: 15-19yrs  sex: 48% female  ethnic group: 85% White | ✓ | ✓ | Social support networks, civic engagement, higher quality schools and neighbourhoods protective. Speaking native language of country migrated to associated with increased risk. Inconsistent findings for role of family social capital. |
| Aim: To understand social influences on adolescent risk-taking (alcohol use, smoking, risky sexual behaviour). | | | | | |
| Wen et al 2012 [41]  China  moderate quality | cross-sectional survey | size: 625  age group: children & adolescents  age range: 8-18 yrs  sex: 49% female  ethnic group: Chinese | ✓ | ✓ | Children/adolescents whose mother has migrated for employment more likely to report health risk behaviours. Support received from the family was protective. No role for community social capital. |
| Aim: To examine the similarities and differences in psychological and behavioural outcomes (smoking, alcohol, drugs) of children living in migrant and non-migrant families. | | | | | |
| **Authors, Country**  **Quality rating** | **Design** | **Sample** | **Assessed**  **FSC CSC** | | **Outcomes** |
| Winstanley et al 2008 [65]  USA  high quality | cross-sectional survey (National Survey of Drug Use and Health) | size: 38,115  age group: adolescents  age range: 12-17 yrs  sex: 49% female  ethnic group: 67% White | ✓ | ✓ | Increased civic participation associated with lower odds of reporting drug/alcohol use and dependence. Increased neighbourhood disorganisation associated with higher odds of reporting drug/alcohol use. Living with only one or neither biological parent was a risk factor. |
| Aim: To examine the influence of self-reported neighbourhood disorganisation and social capital as community factors for adolescent alcohol or drug use dependence | | | | | |
